# Prediction of global potential suitable habitats of *Nicotiana alata* Link et Otto based on MaxEnt model

**DOI:** 10.1038/s41598-023-29678-7

**Published:** 2023-03-24

**Authors:** Yan-Fang Zhang, Shu-Tong Chen, Yun Gao, Long Yang, Hua Yu

**Affiliations:** College of Plant Protection, Shandong Agricultural University, Tai’an, 271018 China

**Keywords:** Ecology, Climate sciences, Ecology

## Abstract

*Nicotiana alata* Link et Otto, widely used in landscaping, is not only of great ornamental value but also of high commercial and medical value. The global potential habitat of *N. alata* and the environmental factors affecting its distribution are not that clear at present. To provide a reference for the reasonable and extensive planting of *N. alata* now and in the future, the MaxEnt model was used to predict its global suitable habitats under current and future climate conditions, respectively, based on global geographic distribution data of *N. alata* and the current and future world bioclimatic variables. The results showed that mean temperature of the driest quarter (bio9), precipitation of driest month (bio14), precipitation seasonality (bio15) and max temperature of warmest month (bio5), were the key bioclimatic variables governing the distribution of *N. alata*. The global suitable habitats of *N. alata* were mainly distributed in Europe, the United States, southeastern South America, and China under current climate conditions. Compared with current climate conditions, the future climate decreased suitable habitats of *N. alata* under SSP1-2.6, and SSP2-4.5 scenario and increased suitable habitats of *N. alata* under SSP3-7.0, and SSP5-8.5 climatic scenarios. The results provided valuable information and theoretical reference for the reasonable planting of *N. alata*.

## Introduction

*Nicotiana alata* Link et Otto, also known as *N. alata*, jasmine tobacco, nicotiana, and sweet tobacco, is a limited perennial herb of the Solanaceae genus *Nicotiana L*. *N. alata* has a variety of flower colors, continuous flowering, bright flower colors, long flowering period, and high ornamental value^1^. It also exists a variety of plant types: the tall type is 45–55 cm high, the middle type is 25–30 cm high, and the common type is shorter only 10–15 cm, therefore *N. alata* can be planted in clusters in curbs, flower beds, parks as greenery, and can be placed on balconies, rooms, and patios to beautify home. *N. alata* has many other uses. Seeds of *N. alata* contain a large amount of fatty acids and nutrients, which have become the raw materials for human health products^2^. Natural flavor compounds with a unique aroma and biological metabolites can be separated from *N. alata*^3^, which can be used in research related to cosmetics and aroma products^4^. *N. alata* can isolate defensins and other substances, which play important roles in defense against fungi and can induce tumor cell death^5–10^. *N. alata* can produce acyl sugars, which have insecticidal and antibacterial properties^11,12^. In recent years, *N. alata* has often been cultivated as a model plant for horticulture, medicine, and scientific research^13^.

*N. alata* prefers deep, fertile, well-drained soil and sunny, warm environment. It is drought tolerant but not cold tolerant. Originating in Argentina and Brazil, *N. alata* is now widely distributed in Europe, North and South America, and Oceania. It is also grown in some parts of China. However, it is not very clear whether there are still suitable areas where *N. alata* has not been introduced, nor whether the areas where *N.alata* have been planted will still be suitable in the future. The prediction of suitable areas for *N.alata* under present and future climate conditions will shed light on these questions.

Species distribution models (SDMs), also known as ecological niche models^14^, are a class of models used to predict the distribution of suitable species habitats based on the study of different scenarios of current and future climate changes. Factors influencing the distribution of species can also be analyzed in detail. SDMs have been widely used in ecology, biogeography, and evolutionary studies^15,16^. With the development of GIS (Geographic Information System) and the application of new statistical techniques, a variety of ecological niche models have been established internationally for the prediction of species-suitable habitats^17^, such as the generalized additive model (GAM), domain model (DOMAIN), bioclimatic model (BIOCLIM), Ecological-niche factor analysis model (ENFA), maximum entropy model (MaxEnt), etc.^18^. Among these models, MaxEnt is the most widely used ecological niche model because of its easy operation, fast running speed^19^, and accurate prediction of the potentially suitable habitats of species based on the longitude and latitude of known presence points and climate factors or even small sample data. MaxEnt has been widely used in invasion management of biological species^20–22^, protection of endangered plants and animals^23–26^, control of pests and diseases^19^, and environmental risk assessment of species habitats^27^.

At present, scholars about *N. alata* at home and abroad had focused on the expression pattern analysis of essential genes^1,28,29^, cultivation management^30–33^ and characteristics of fertilizer requirements, self-incompatibility mechanism, and regeneration system^29,34–36^, etc. No research has been done on the distribution and growing areas of *N. alata*. Here, to provide a reference for the planting of *N. alata*, MaxEnt ecological niche model was used to predict the range of suitable planting areas for *N. alata* under current and future climate conditions. This study will reveal the suitable areas of *N. alata* at present and predict possible changes of suitable areas in the future, and hence provide a theoretical basis for the reasonable planting of *N. alata.*

## Materials and methods

### Global sample sites of *N. alata*

Records of the presence of *N. alata* were obtained from the Global Biodiversity Information Facility (GBIF, https://www.gbif.org/)^37^, the Chinese Virtual Herbarium (CVH, https://www.cvh.ac.cn/), the National Specimen Information Infrastructure (NSII, http://nsii.org.cn/2017/AboutUs.php), and Chinese Field Herbarium (CFH, https://cfh.ac.cn/). Then, these sample points were screened to ensure the correct information of *N. alata* data. Firstly, sample points with correct Latin names and accurate latitude and longitude were selected, and obviously, wrong geocoded data and duplicate data were excluded. Secondly, sample points with detailed geographic locations but no latitude and longitude information were located using Google Earth to obtain accurate latitude and longitude information. Finally, 1023 sample points of *N. alata* were obtained.

**Figure 1:**
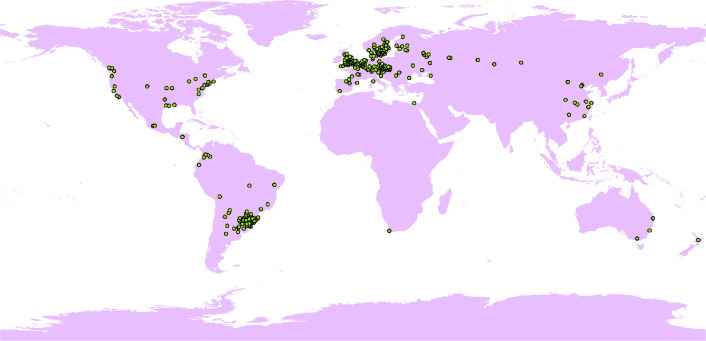
Global occurrence records used for the model simulation to predict the global potential suitable habitats of *N.alata*. This base map was directly obtained from the calculation of MaxEnt with the bioclimatic data from WorldClim. Global bioclimate data were acquired from the WorldClim open database (https://worldclim.org). The locations of species occurrence were collected from open databases: GBIF (http://www.gbif.org), the Chinese Virtual Herbarium (CVH, https://www.cvh.ac.cn/), the National Specimen Information Infrastructure (NSII, http://nsii.org.cn/2017/AboutUs.php), and Chinese Field Herbarium (CFH, https://cfh.ac.cn/). The species distribution model was conducted with MaxEnt (Version 3.4.4, http://biodiversityinformatics.amnh.org/open_source/maxent/), and results were modified with ArcGIS (version 10.7, https://www.arcgis.com/).

Distributional data are usually non-random and the datasets are hardly used for systematic studies, therefore the datasets that emerge have strong geographical bias and spatial autocorrelation, which can lead to spurious model results. Using the Thin function of the R package spThin, a refinement distance of 50 km was chosen to spatially dilute the dataset of *N. alata*^38^, resulting in 300 sample points of *N. alata* (Fig. 1).

### Environmental variable data

Climate variables are the main determinants of species' ecological niches in the study of species distributions at large spatial scales^39^. The current bioclimatic data used in this study were obtained from the WorldClim 2.1 climate database (https://www.worldclim.org/data/worldclim21.html) with a spatial resolution of 2.5 min. These bioclimatic variables are recorded as mean values from 1970 to 2000 and represent annual mean temperature and precipitation trends, seasonal trends, and extreme trends.

To better predict the potential future suitable habitats of *N. alata*, four shared socio-economic pathways (SSPs), SSP1-2.6, SSP2-4.5, SSP3-7.0, and SSP5-8.5, were selected from the moderate resolution National (Beijing) Climate Center climate system model (BCC-CSM2-MR) for the year 2070 (2061–2080 average) of the Coupled Model Intercomparison Project Phase 6 (CMIP6)^40^. Select the bioclimatic variables (bc) from the four shared social pathways, import the downloaded bioclimatic variables into ArcGIS, and use the "raster conversion tool" to convert the .tif format of 19 bioclimatic variables into .asc format for use in the model. The shared socioeconomic pathway examines different future greenhouse gas emissions, providing a range of different end-of-century climate change outcomes. Each scenario has similar 2100 radiative forcing levels as its predecessor in the Fifth Assessment Report (AR5). SSP1-2.6 is the sustainable development pathway with warming limited to less than 2 °C, SSP2-4.5 is a moderate development path with warming limited to less than 3 °C, SSP3-7.0 is the partial development pathway with warming limited to less than 4.1 °C, and SSP5-8.5 is the conventional development path with warming limited to less than 5 °C.

During the model run, the covariance between bioclimatic variables can exist, resulting in the overfitting of the model. To reduce the covariance of bioclimatic variables, multiple covariances were eliminated by estimating Pearson correlation coefficients and variance inflation factors (VIF) for two-by-two comparisons of 19 bioclimatic variables. Variables with VIF less than 10 and |r| ≤ 0.70 for each correlation were considered as the final predictor variables of the model^41,42^, and seven bioclimatic variables were finally selected (Table 1).

**Table 1:** The seven bioclimatic variables used for the MaxEnt model.

Bioclimatic variables	Variable description	Unit
bio14	Precipitation of Driest Month	mm
bio9	Mean Temperature of Driest Quarter	°C
bio7	Temperature Annual Range	°C
bio15	Precipitation Seasonality	–
bio5	Max Temperature of Warmest Month	°C
bio2	Mean Diurnal Range	°C
bio18	Precipitation of Warmest Quarter	mm

### Model simulation

MaxEnt model software (version 3.4.4) was downloaded from https://biodiversityinformatics.amnh.org/open_source/maxent/ for maximum entropy simulations to predict suitable habitats of *N. alata*^43^*.* ArcGIS 10.7, a comprehensive GIS invented by ESRI (Environmental Systems Research Institute, Inc), was used for the analysis of layers, the transformation of data formats, and reclassification of results.

**Figure 2:**
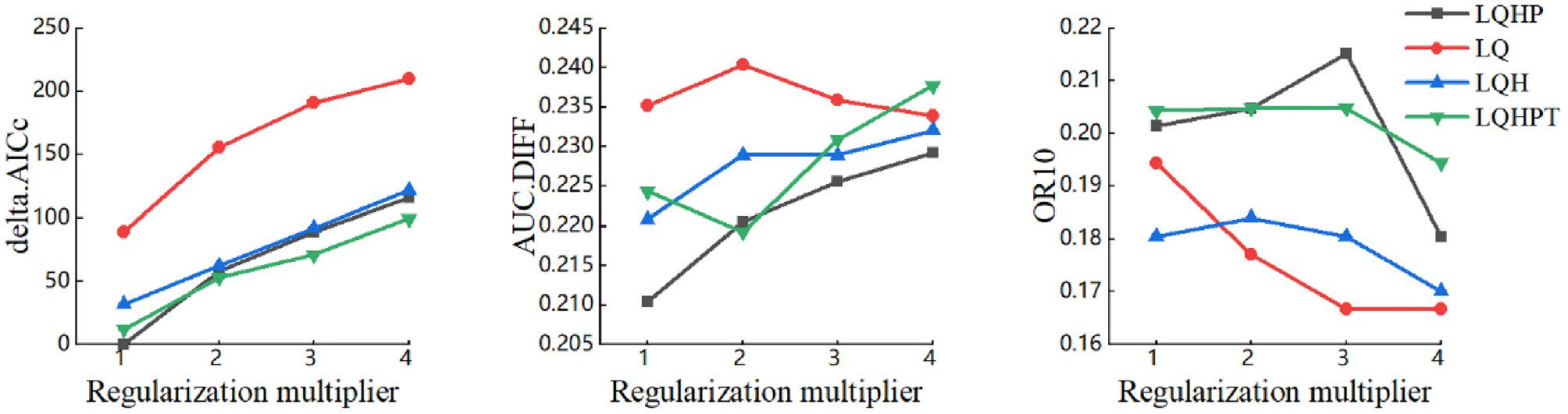
Assessment metrics of MaxEnt generated by ENMeval.

In the MaxEnt model-based prediction of the potential geographic distribution of species, although most researchers use default parameters in their modeling, some researchers have noted that the relative complexity of models constructed using default parameters can lead to overfitting, which makes the results difficult to interpret^44^. In this paper, we use the R program package ENMeval^45^ to adjust the regularization multipliers (RM) and feature combinations (FC)^46^, and analyze the complexity of the model with different parameter combinations in order to select the combination with lower complexity for modeling and optimize the model. In this study, the RM parameters were set to 1–4 with an interval of 1 for each, for a total of four RM parameters. For the FC parameters, the MaxEnt model provides five features: linear (L), quadratic (Q), hinge (H), product (P), and threshold (T). We chose four combinations of features: LQ, LQH, LQHP, and LQHPT. The Akaike information criterion correction (AICc) was used to evaluate the fit and complexity of the model; the difference between training and testing AUC (AUC.DIFF) and 10% training omission rate (OR10) were used to evaluate the degree of overfitting of the model^41^. The parameter combination had the minimum delta.AICc value had been chosen as our optimal parameter to build the model^41^. Based on the lambdas file, RM was set to 1 and FC was set to LQHP, which was the best combination for the MaxEnt model in this study (Fig. 2).

Import the respective data of global sample points and bioclimatic variables of *N. alata* into MaxEnt software. Run linear features, quadratic features, hinge features, and product features. Then, 75% of the randomly selected occurrence records were used as the training set with the remaining 25% as the test set to construct the MaxEnt prediction model^47^. For each training partition, 10 replications were generated and the results were averaged after 500 iterations at the end^48^. Response curves and jackknife were used to analyze the effects of bioclimatic variables on species distribution. The accuracy of the MaxEnt prediction model was analyzed using receiver operating characteristic (ROC) curves. The final software output file format was logistic and the output file type was ASC.

### Model performance evaluation and area calculation

Three methods were used to evaluate the model: the area under the receiver operating characteristic curve (AUC)^49,50^, the continuous Boyce index (CBI)^51^, and true skill statistics (TSS)^49^.

The receiver operating characteristic (ROC) curve, widely used in the prediction of the potential suitable habitats of species^30^, is a highly recognized parameter for the evaluation of diagnostic tests. The area under the ROC curve is AUC^52^ (Area Under Curve) which is used to analyze the model accuracy, with AUC values ranging from 0 to 1. When AUC > 0.9, the prediction is very accurate. TSS^52^ is an intuitive threshold correlation measure with values ranging from − 1 to + 1, where 1 indicates perfect model performance and values of zero or less indicate poor performance^49^. When TSS > 0.75, the model prediction is excellent^53^. CBI values vary between − 1 and + 1 as well, for which the positive values indicate that the model prediction distribution matches the observed presence data, values near to zero indicate random model predictions, and negative values indicate inverse predictions^51^.

Import the model results into ArcGIS to a raster format, project the raster data (WGS1984), classify and visualize the simulated distribution into suitable habitats. The 10th percentile training presence as the suitability threshold was applied to build a potential suitability map^54,55^. We imported reclassified data into four classes of habitat suitability, lowly suitable habitat (threshold–0.40 probability of occurrence), moderately suitable habitat (0.40–0.60 probability of occurrence), high suitable habitat (> 0.60 probability of occurrence), and unsuitable habitat (0–threshold probability of occurrence). The raster file was converted into a polygon file by using the raster-to-raster conversion tool in ArcGIS and then the calculation function in ArcGIS properties was used to calculate the area of each suitable habitat and determine the proportion of each area. We converted the current and future output raster files of *N. alata* to presence-absence (1/0) format using the merged SDMToolbox in ArcGIS, subtracted the binary mapping of the future scenario from the current (baseline) binary map to account for range changes between the current and future models.

## Results

### Accuracy analysis of the MaxEnt prediction model for the global suitable habitats of *N. alata*

In order to characterize the prediction accuracy of the obtained MaxEnt prediction model, AUC, TSS and CBI were calculated. The model achieved the highest predictive accuracies and had the highest performance, obtaining AUC of 0.944, TSS of 0.759, and CBI of 0.992. This indicated that the prediction accuracy of the obtained MaxEnt prediction model was high and the model would be very accurate in predicting the global suitable habitats of *N. alata*.

### Contribution of each bioclimatic variable to the MaxEnt prediction model

The further calculation was performed to characterize the contribution of each bioclimatic variable to our MaxEnt prediction model. The results showed that among the seven bioclimatic variables used to construct the MaxEnt prediction model (Table 2), the top three contributors were precipitation of driest month (bio14), mean temperature of driest quarter (bio9), and temperature annual range (bio7), with a cumulative contribution of 82.2%. The total contribution of the remaining four bioclimatic variables was 17.8%. Overall, precipitation of driest month (bio14), mean temperature of driest quarter (bio9), and temperature annual range (bio7) were the main bioclimatic variables that had the greatest impact on the MaxEnt prediction model.

**Table 2:** Contribution of the seven bioclimatic variables to the MaxEnt prediction model.

Variables	Contribution (%)
Precipitation of Driest Month (bio14)	51
Mean Temperature of Driest Quarter (bio9)	16.1
Temperature Annual Range (bio7)	15.1
Precipitation Seasonality (bio15)	7.9
Max Temperature of Warmest Month (bio5)	7
Mean Diurnal Range (bio2)	1.5
Precipitation of Warmest Quarter (bio18)	1.5

### Contribution of each bioclimatic variable to the distribution of* N. alata*-jackknife test of the MaxEnt prediction model

**Figure 3:**
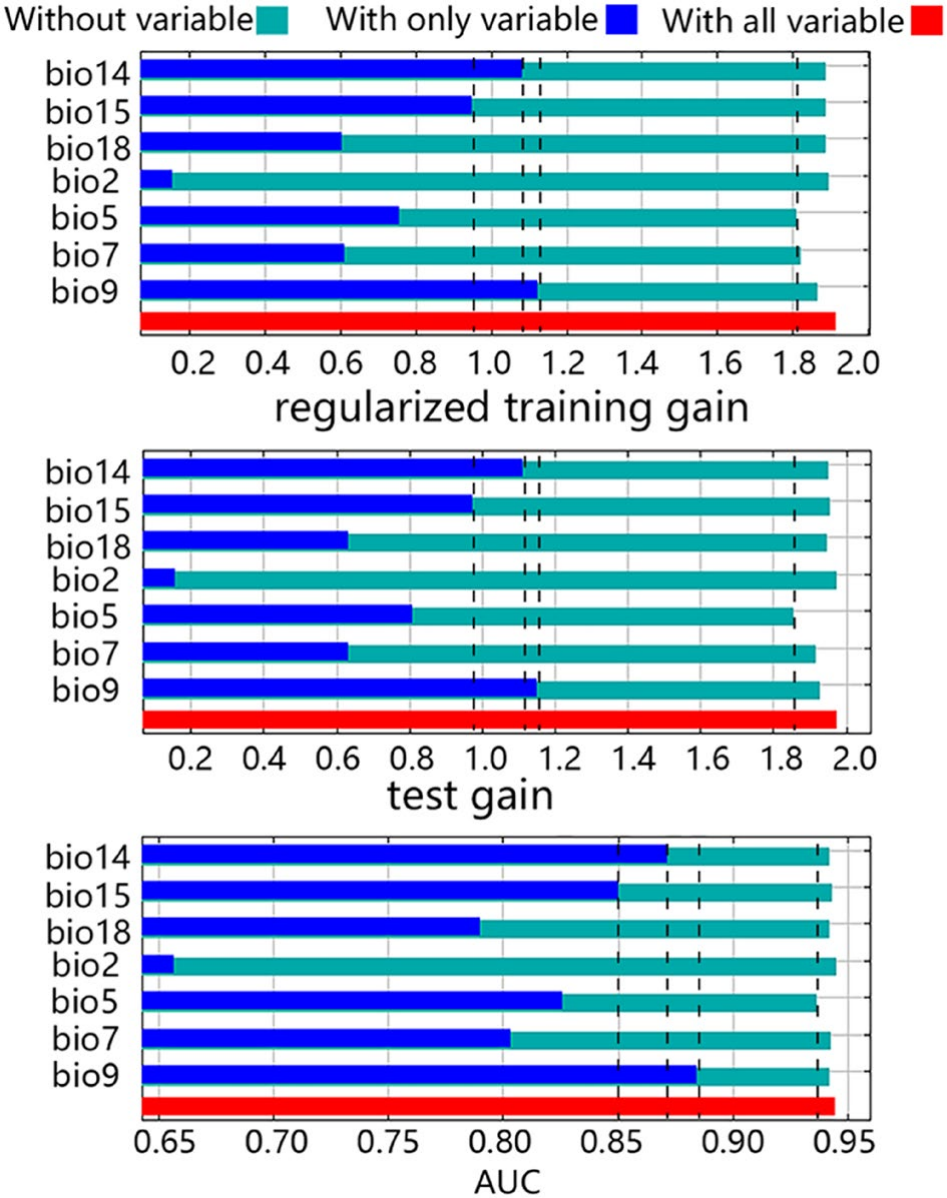
The jackknife test of the MaxEnt prediction model for the prediction of the global suitable habitats of *N. alata*.

The jackknife test was used to estimate the contribution of each bioclimatic variable to the distribution of *N. alata* based on the performance of our MaxEnt prediction model for the global suitable habitats of *N. alata*. From the results of the test with only variable (Fig. 3, blue bars), the top three bioclimatic variables in regularized training gain, AUC, and test gain were all mean temperature of the driest quarter (bio9), precipitation of driest month (bio14), and precipitation seasonality (bio15), implying the importance of these three bioclimatic variables to the distribution of *N. alata,* since the longer the bar, the more important the bioclimatic variable was for the species distribution. The results of the test without variable (Fig. 3, green bars) showed that max temperature of warmest month (bio5) had the most reduced values in test gain, AUC, and regularized training gain, indicating that this bioclimatic variable contained much more specific environmental information in the prediction of the distribution of *N. alata*, as the green bar represented the specificity of the variable, and the shorter the bar, the more special information the bioclimatic variable contained that was not present in other variables. Additionally, the red bar represented the performance of the model with all seven bioclimatic variables involved, which acted as a control group for other tests. In summary, the results of the jackknife analysis indicated that mean temperature of the driest quarter (bio9), precipitation of driest month (bio14), precipitation seasonality (bio15), and max temperature of warmest month (bio5) were the key bioclimatic variables governing the distribution of *N. alata*.

**Figure 4:**
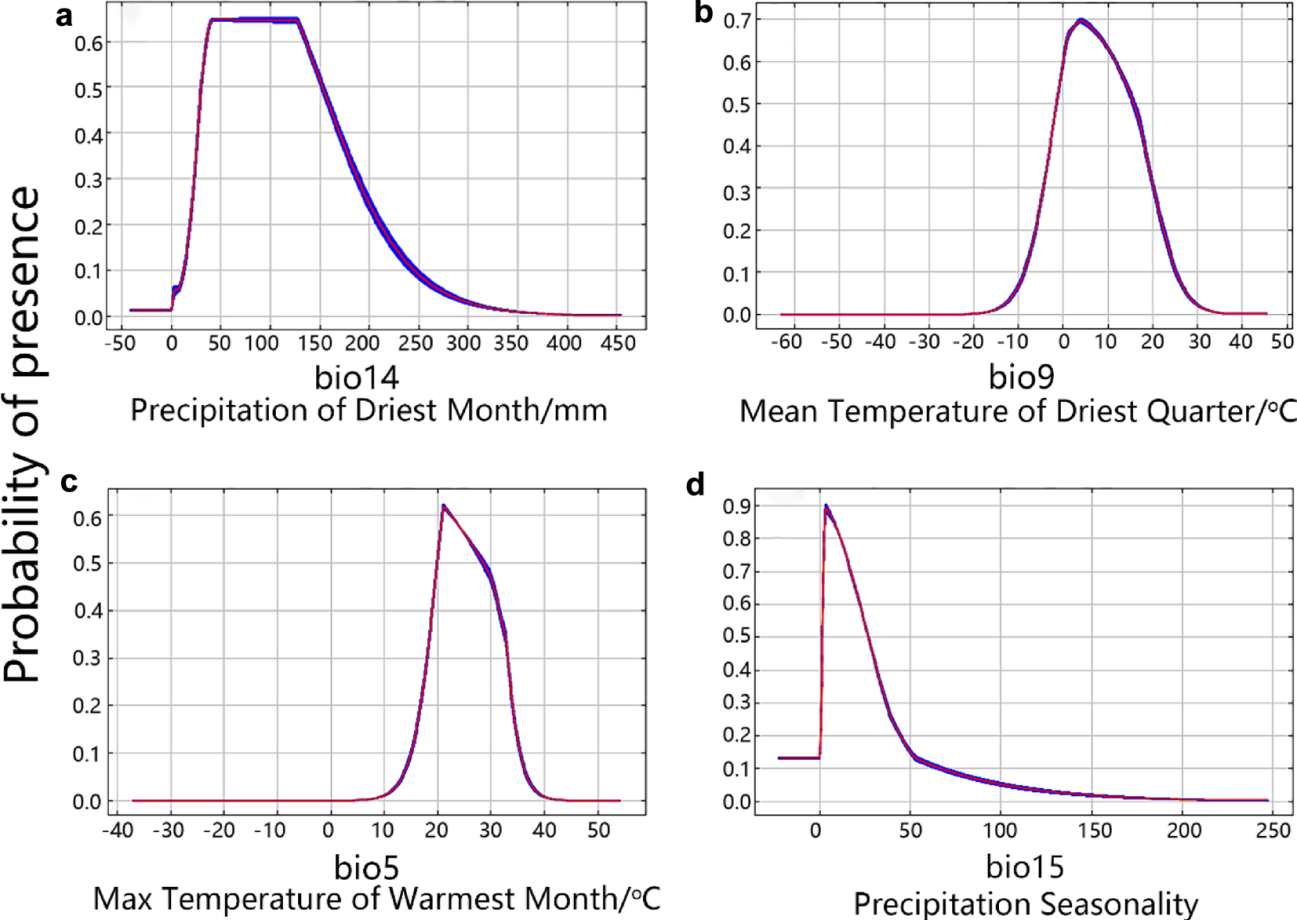
Response curve showing the relationships between the probability of the presence of *N. alata* and the key four bioclimatic variables. (**a**) response curve of *N. alata* to precipitation of driest month (bio14), (**b**) response curve of *N. alata* to mean temperature of driest quarter (bio9), (**c**) response curve of *N. alata* to max temperature of warmest month (bio5), (**d**) response curve of *N. alata* to precipitation seasonality (bio15). The probability values shown are the average over 10 replicate runs. Blue margins show ± SD (standard deviation) calculated over 10 replicates.

To better cultivate *N. alata*, the optimum value range of the above four key bioclimatic variables, obtained from the results of the jackknife test, were further explored. 0.6–1 was used to determine the optimum value range of each variable (Fig. 4), which was also the determinant of highly suitable habitats. As a result, all four bioclimatic variables obtained an optimum value range, in which *N. alata* grows well, from their respective response curve (Fig. 4). The optimum value range of precipitation of driest month (bio14) was 38–127 mm, with whole suitable range of 15–207 mm in which *N. alata* can grow (Fig. 4a). The optimum value range of mean temperature of driest quarter (bio9) was 0–11 °C, with the probability of *N. alata* presence increased from − 7 °C to 3 °C and decreased from 3 to 21 °C (Fig. 4b). The optimum value of max temperature of warmest month (bio5) was 20.2–23 °C, with the probability of *N. alata* presence increased from 18 to 21 °C and decreased from 21 to 32 °C (Fig. 4c). The optimum value range of precipitation seasonality (bio15) was 1–25, and the probability of presence of *N. alata* decreased at 3–47 (Fig. 4c).

### Prediction of the global potential suitable habitats of *N. alata* under current climate conditions

**Figure 5:**
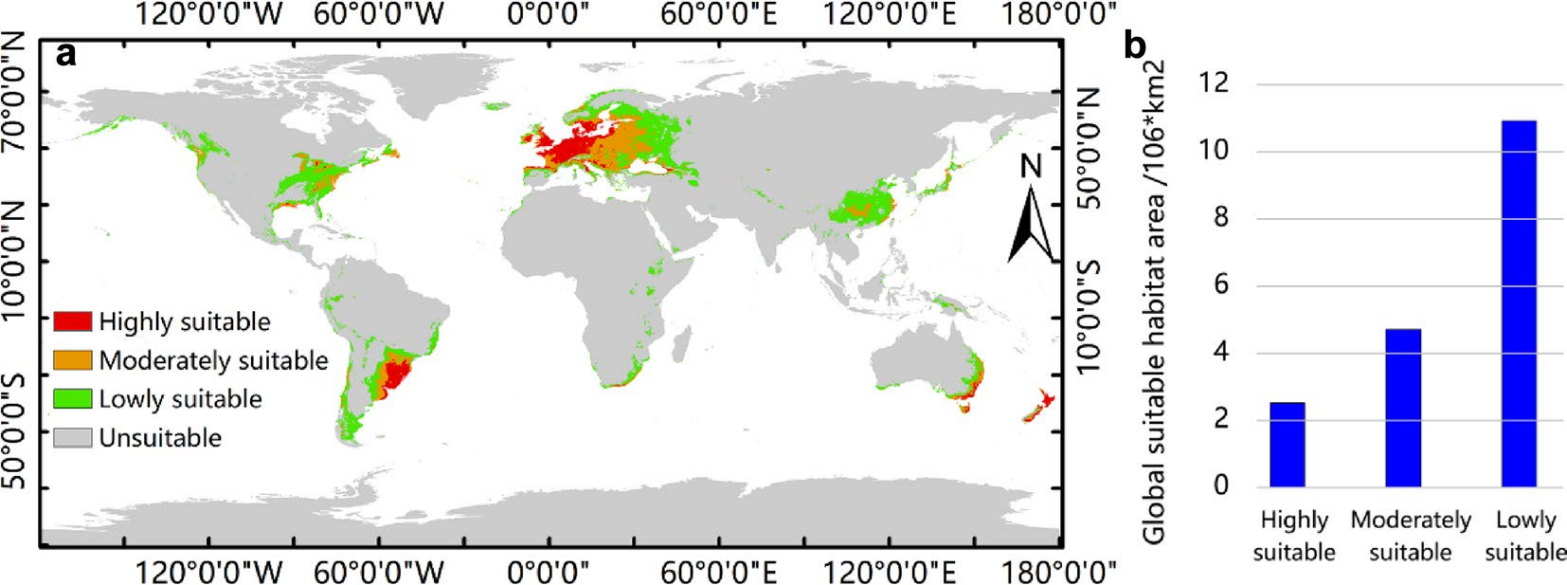
The predicted global suitable habitats of *N. alata* under current climate conditions. (**a**) global suitable habitats of *N. alata*, (**b**) global suitable habitat areas of *N. alata.* This base map was directly obtained from the calculation of MaxEnt with the bioclimatic data from WorldClim. Global bioclimate data were acquired from the WorldClim open database (https://worldclim.org). The locations of species occurrence were collected from open databases: GBIF (http://www.gbif.org), the Chinese Virtual Herbarium (CVH, https://www.cvh.ac.cn/), the National Specimen Information Infrastructure (NSII, http://nsii.org.cn/2017/AboutUs.php), and Chinese Field Herbarium (CFH, https://cfh.ac.cn/). The species distribution model was conducted with MaxEnt (Version 3.4.4, http://biodiversityinformatics.amnh.org/open_source/maxent/), and results were modified with ArcGIS (version 10.7, https://www.arcgis.com/).

The MaxEnt prediction model obtained above was used to predict the potential suitable habitats of *N. alata* under current climate conditions. The results showed that the highly suitable habitats of *N. alata* were mainly located in Germany, France, England, Uruguay, southern Brazil, New Zealand, the Netherlands, Belgium, Denmark, and eastern Australia, and so on (Fig. 5a). The moderately suitable habitats were located in Poland, the Czech Republic, Slovakia, Hungary, Belarus, Lithuania, Serbia, Austria, Slovenia, Croatia, Ukraine, southern France, northern Italy, Paraguay, and Argentina, and so on (Fig. 5a). The lowly suitable habitats were located in Macedonia, central Romania, northern Russia, southern Finland, western Russia, the United States, and other regions (Fig. 5a). Among them, the area of highly suitable habitats was 2.52 × 10^6^ km^2^, accounting for 13.88% of the total suitable habitat area (Fig. 5b). The area of moderately suitable habitats was 4.7 × 10^6^ km^2^, accounting for 25.88% of the total suitable habitat area (Fig. 5b). The area of lowly suitable habitats was 10.94 × 10^6^ km^2^, accounting for 60.24% of the total suitable habitat area (Fig. 5b).

### Prediction of the global potential suitable habitats of *N. alata* under future climate conditions

**Figure 6:**
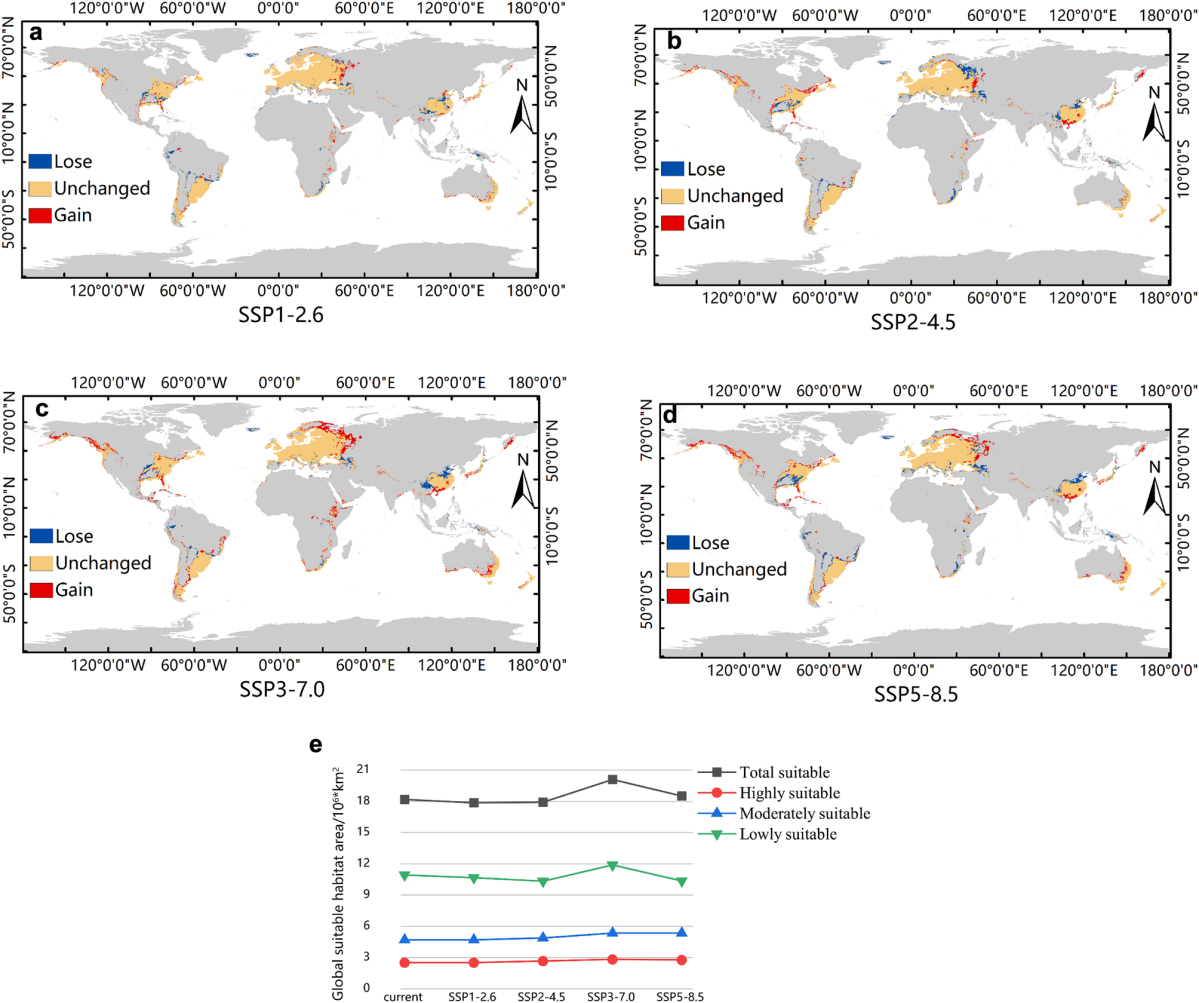
Changes of the global suitable habitats of *N. alata* between different future climate scenarios and the current climate conditions. (**a**) the global suitable habitats changes of *N. alata* between the future SSP1-2.6 climate scenario and the current climate conditions, (**b**) the global suitable habitats changes of *N. alata* between the future SSP2-4.5 climate scenario and the current climate conditions, (**c**) the global suitable habitats changes of *N. alata* between the future SSP3-7.0 climate scenario and the current climate conditions, (**d**) the global suitable habitats changes of *N. alata* between the future SSP5-8.5 climate scenario and the current climate conditions, (**e**) area line chart of suitable habitats of *N. alata* under current climate condition and four future climate scenarios. This base map was directly obtained from the calculation of MaxEnt with the bioclimatic data from WorldClim. Global bioclimate data were acquired from the WorldClim open database (https://worldclim.org). The locations of species occurrence were collected from open databases: GBIF (http://www.gbif.org), the Chinese Virtual Herbarium (CVH, https://www.cvh.ac.cn/), the National Specimen Information Infrastructure (NSII, http://nsii.org.cn/2017/AboutUs.php), and Chinese Field Herbarium (CFH, https://cfh.ac.cn/). The species distribution model was conducted with MaxEnt (Version 3.4.4, http://biodiversityinformatics.amnh.org/open_source/maxent/), and results were modified with ArcGIS (version 10.7, https://www.arcgis.com/).

The MaxEnt prediction model obtained above was used to predict the global potential suitable habitats of *N. alata* under future climate conditions. The predictions showed that the global potential habitats of *N. alata* under the four different scenarios of future climate conditions (Fig. S1) were slightly different from the prediction result under the current climate conditions (Fig. 6a–d). In the SSP1-2.6 scenario, the total area of the global suitable habitats of *N. alata* was 17.87 × 10^6^ km^2^, which was 2.9 × 10^5^ km^2^ less than that under the current conditions (Fig. 6e). This area decrease was not simply a loss of old suitable habitats, it occurred with occurrence of some new suitable habitats (Fig. 6a, red) and more loss of old ones (Fig. 6a, blue). The areas of highly, moderately and lowly suitable habitats were 2.51 × 10^6^ km^2^, 4.7 × 10^6^ km^2^ and 10.66 × 10^6^ km^2^, respectively. The highly and lowly suitable habitats decreased compared with the current conditions, while the moderately suitable habitat increased slightly (Fig. 6e). Under the SSP2-4.5 scenario, the total area of the global suitable habitats of *N. alata* was 17.90 × 10^6^ km^2^, which was 2.6 × 10^5^ km^2^ less than that under the current conditions (Fig. 6e), also with occurrence of some new suitable habitats and more loss of old ones (Fig. 6b) just like the situation in the SSP1-2.6 scenario. The areas of the highly, moderately, and lowly suitable habitats were 2.68 × 10^6^ km^2^, 4.88 × 10^6^ km^2^ and 10.34 × 10^6^ km^2^, respectively. Compared with the current conditions, the highly and moderately suitable habitats all showed an expansion trend, at the same time, the lowly suitable habitat had a larger decrease (Fig. 6e). Under the SSP3-7.0 scenario, the total area of the global suitable habitats of *N. alata* was 20.08 × 10^6^ km^2^, which was 1.92 × 10^6^ km^2^ more than that under the current environment (Fig. 6e). Similarly, this area increase occurred with some loss of old suitable habitats (Fig. 6c, blue) and the emergence of more new suitable habitats (Fig. 6c, red). The areas of highly, moderately, and lowly suitable habitats were 2.84 × 10^6^ km^2^, 5.35 × 10^6^ km^2^, and 11.89 × 10^6^ km^2^, respectively, which all expanded compared with the corresponding area under the current conditions (Fig. 6e). Under the SSP5-8.0 scenario, the total area of the global suitable habitats of *N. alata* was 18.51 × 10^6^ km^2^, which was 3.5 × 10^5^ km^2^ more than that under the current environment, also with some loss of old suitable habitats (Fig. 6d, blue) and the emergence of more new suitable habitats (Fig. 6d, red). The areas of highly, moderately, and lowly suitable habitats were 2.79 × 10^6^ km^2^, 5.35 × 10^6^ km^2^, and 10.37 × 10^6^ km^2^. Compared with the current conditions, the highly and moderately suitable habitats all showed an expansion trend, while the lowly suitable habitat showed a slightly reduction (Fig. 6e).

## Discussion

*N. alata* is a well-known ornamental flower. Detailed knowledge of its distribution is a prerequisite for its rational distribution and utilization in an ecosystem. This study provides a detailed analysis of the potential global habitats of *N. alata* under current and future climate conditions based on the MaxEnt model. Our results provide a theoretical basis for the development of practical planting and management measures for *N. alata*.

Temperature and precipitation have important effects on species distribution, however, each specific bioclimatic variable may have different effects on different species owing to the differences in the species' growth habits. In this study, the results showed that mean temperature of the driest quarter (bio9), precipitation of driest month (bio14), precipitation seasonality (bio15), and max temperature of warmest month (bio5) were the key bioclimatic variables that affect the growth of *N. alata*. *N. alata* grows in a warm and sunny environment and is not cold tolerant, too low temperature tends to freeze the root system, resulting in the death of *N. alata*. In this study, max temperature of warmest month (bio5) showed that the temperature suitable for the growth of *N. alata* was 17–32 °C, and below 10 °C, the probability of *N. alata* growth was 0 (Fig. 4c). On the other hand, precipitation affects seedling survival, growth, and plant growth over the full life span. Excess water in the soil can disrupt the water balance required by plants, which can negatively affect their metabolism and morphology, limiting their growth or even leading to death. In this study, we could find that *N. alata* had a very low precipitation requirement based on the precipitation of driest month (bio14) and precipitation seasonality (bio15), and the maximum rainfall in the driest month was 200 mm (Fig. 4a), and more than 200 mm would not be suitable for the growth of *N. alata*. However, each specific bioclimatic variable affects different species differently because of differences of the species' own growth habits. For example, in a prediction of the suitable habitats of Sapin*dus mukorossi* using the MaxEnt model, precipitation of warmest quarter (bio18), min temperature of coldest month (bio6), temperature seasonality (bio4), and isothermality (bio3) were found to be the main bioclimatic variables affecting *Sapindus mukorossi*^18^. The analysis of the suitable habitats of *Osmanthus fragrans* using the MaxEnt model found that UV-B seasonality, precipitation seasonality (bio15), temperature annual range (bio7) and mean diurnal temperature range (bio2) were the most important factors interpreting the environmental demands of this species^48^. The prediction of suitable habitats of *Pinus densiflora* using MaxEnt model revealed that warmest seasonal precipitation (bio18), seasonality of temperature (bio4), mean annual temperature (bio1) and mean temperature of wettest season (bio8) were the main environmental variables affecting the growth of *Pinus densiflora*^56^. Thus, it is clear that the contribution of bioclimatic variables to specific species needs to be studied specifically, and the process of this paper provides a reference and comparison for other species.

The prediction results of this study provide a reference for *N. alata* planting in China. Currently, the presence of *N. alata* is recorded in Inner Mongolia, Heilongjiang, Guangxi, Fujian, Chongqing, Jiangsu, Shanghai, Hubei, and Zhejiang provinces and cities in China. According to the model prediction results (Fig. 5a), the suitable habitats of *N. alata* under current climate conditions cover 2.3 × 10^6^ km^2^ in China, and in addition to the above-known distribution areas of *N. alata*, Shanxi, Guizhou, Guangdong, Yunnan, Sichuan, and Shandong are also suitable for *N. alata* growth, providing a wider planting area for *N. alata* cultivation. Besides, only climatic factors affecting the growth of *N. alata* were considered in this study, other factors such as human activities, variety selection, and soil type, were not considered, but all factors should be considered in the actual introduction of *N. alata* in a certain area to ensure the quality of flowering.

Global climate change has been reported to affect the distribution of various species. Climate change may lead to regional or local extinction of *N. alata* in certain areas as has been predicted in our results. For the conservation of *N. alata*, three main recommendations are proposed. (1) Protect or introduce *N. alata* according to the predicted results under different climate conditions and the actual local conditions. (2) Track and monitor the actual growth of *N. alata*, and then make reasonable planting plans based on the predicted results and the actual conditions, especially for regions where the planting areas of *N. alata* are likely to change according to predictions here. (3) It is necessary to strengthen systematic research on germplasm resources to increase the quality of *N. alata*, since researches on *N. alata* cultivation, growth environment and variety improvement are less at present.

## Supplementary Information

Supplementary Figure S1.

## Data Availability

Data from the current study are available from the corresponding author upon reasonable request.
