# Supplementary material for: Prediction of global potential suitable habitats of Nicotiana alata Link et Otto based on MaxEnt model
Source: Sci Rep. 2023 Mar 24;13:4851. doi: 10.1038/s41598-023-29678-7 (PMC10038996; doi:10.1038/s41598-023-29678-7)
Supplement: Supplementary file 1 — Supplementary Figure S1. [file 41598_2023_29678_MOESM1_ESM.docx]

***Supporting Information***

Prediction of global potential suitable habitats of *Nicotiana alata* Link et Otto based on MaxEnt model

Yan-Fang Zhang^1^, Shu-Tong Chen^1^, Yun Gao^1^, Long Yang^1*^, Hua Yu^1*^

^1^College of plant protection, Shandong Agricultural University, Tai’an 271018, China

Correspondence: lyang@sdau.edu.cn

yuh@sdau.edu.cn


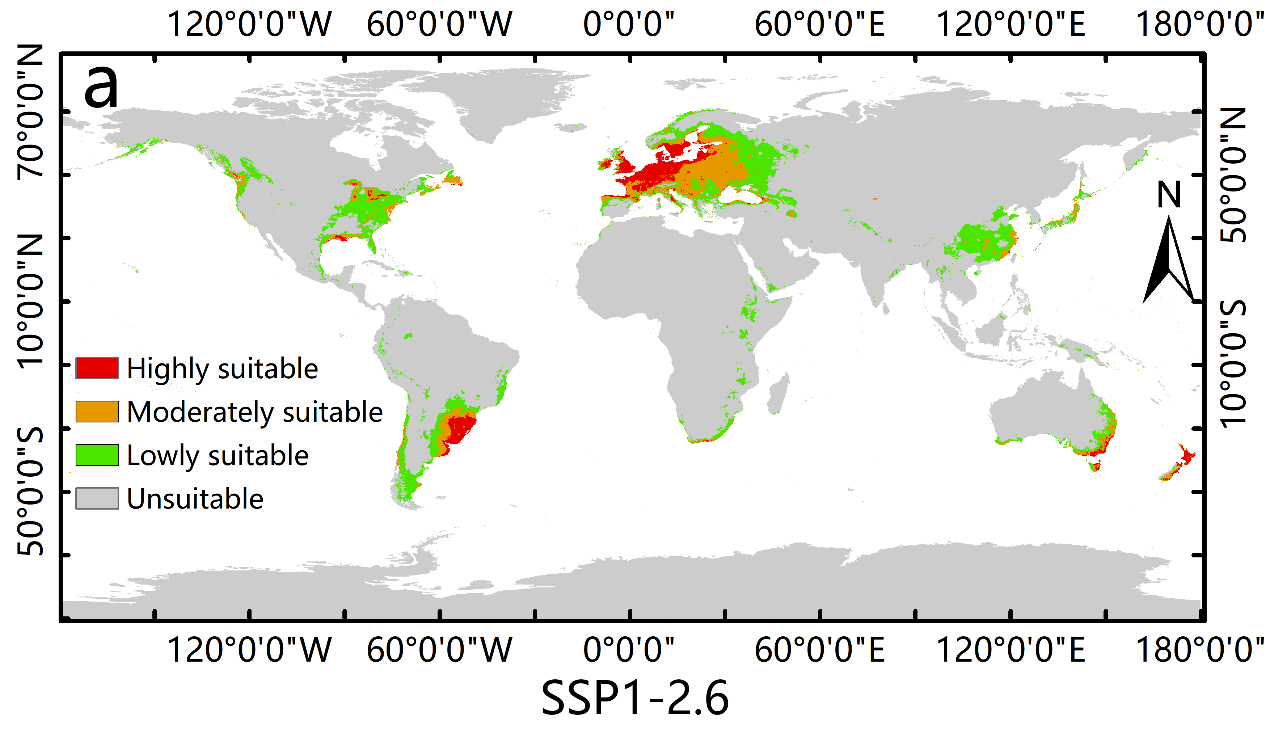

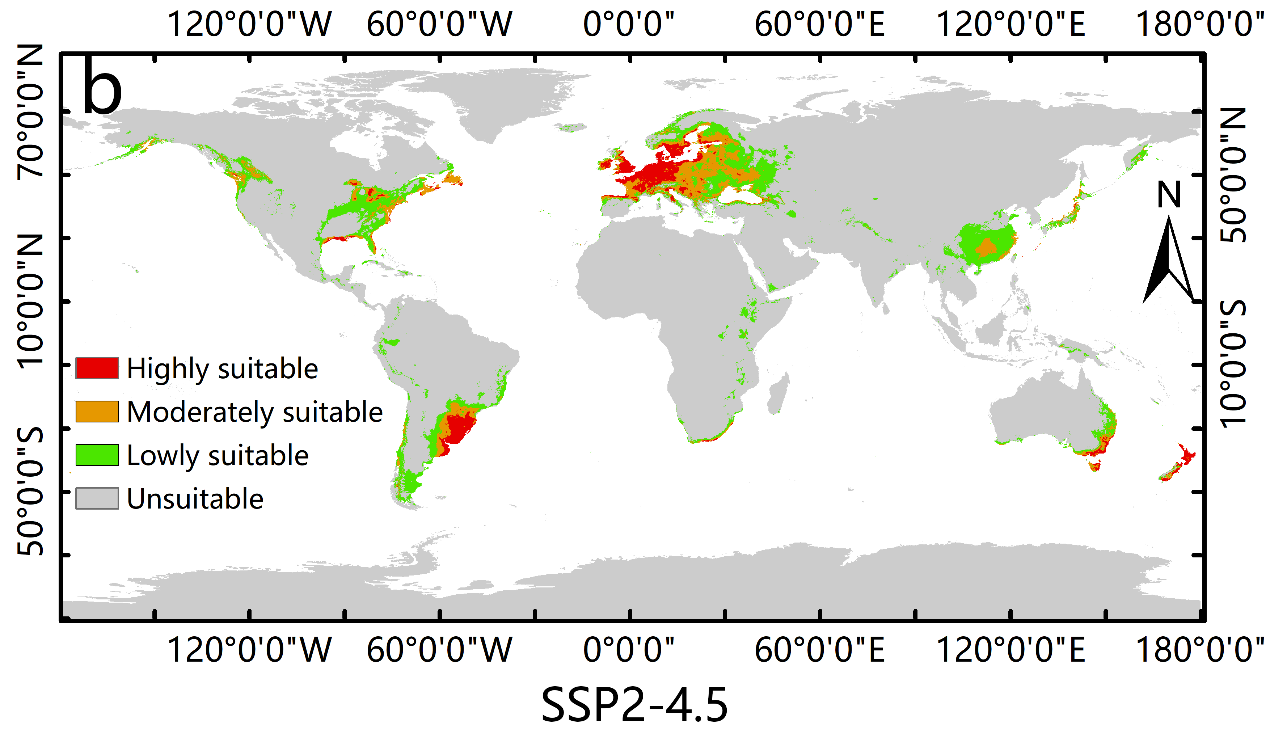

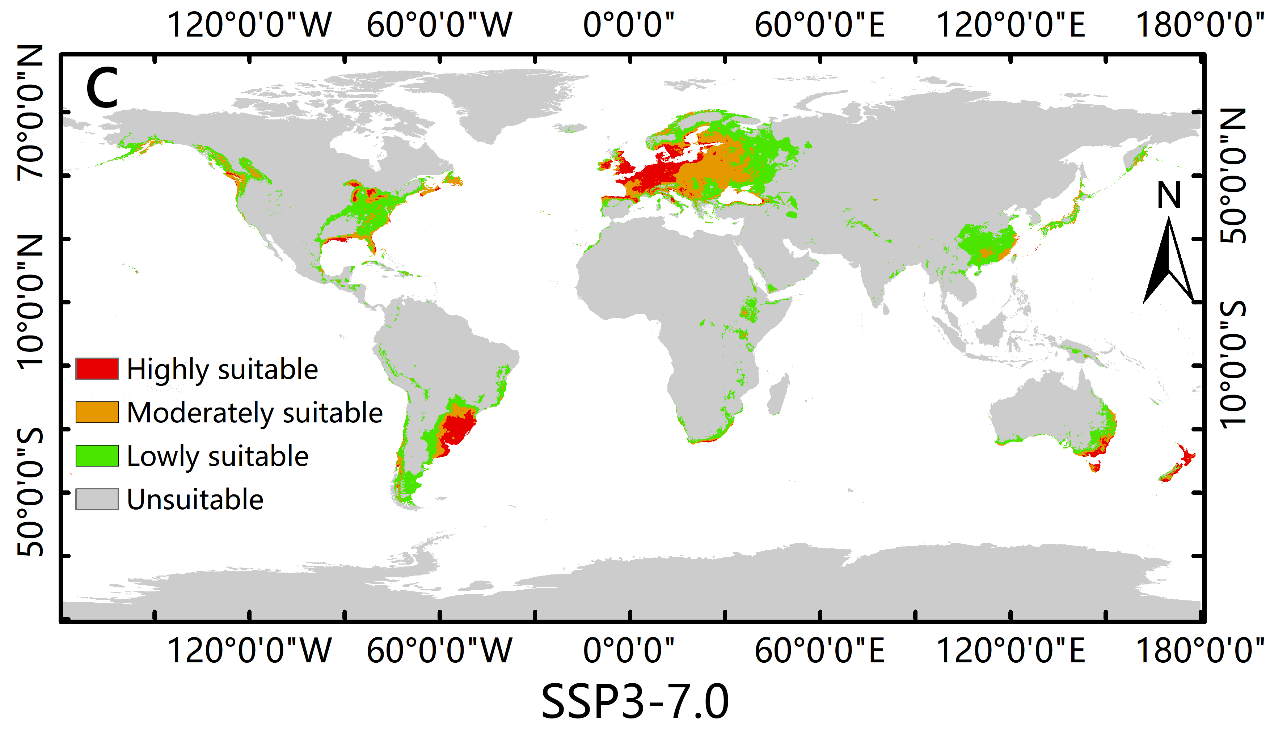

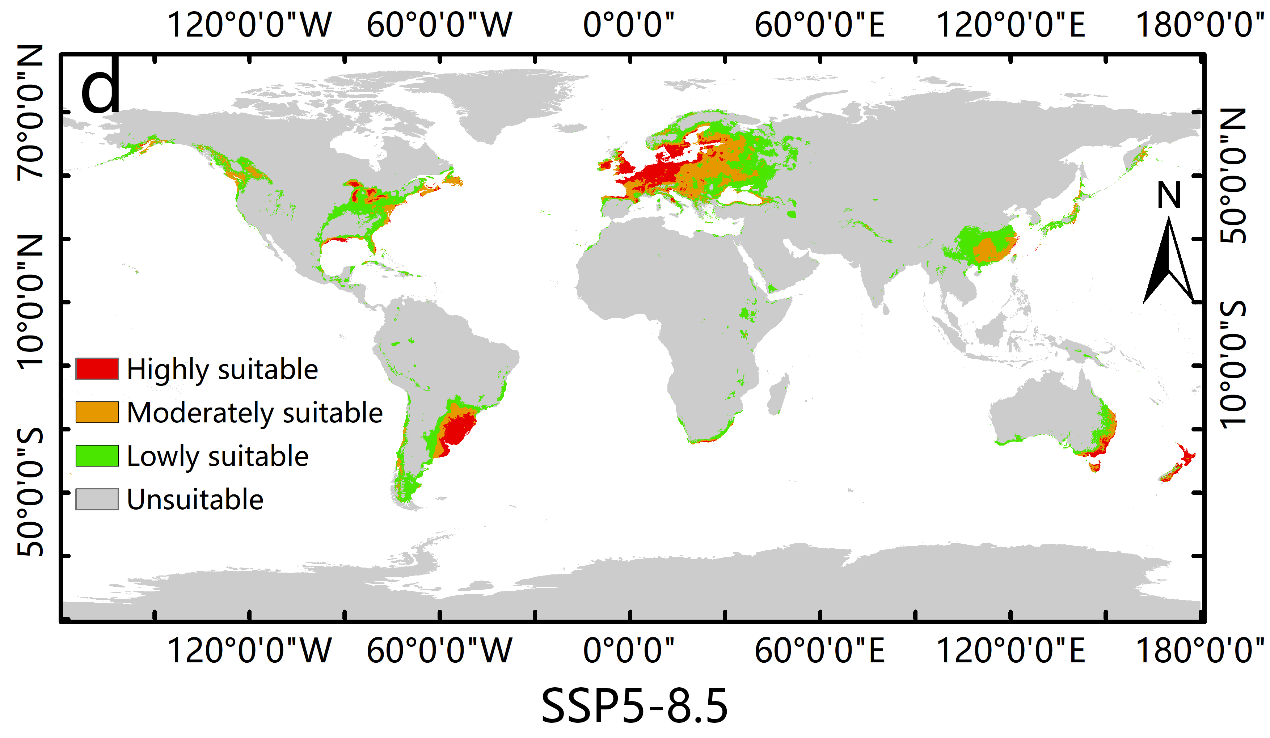


Figure S1 The predicted global suitable habitats of *N. alata* under four different future scenarios. (a) SSP1-2.6 climate scenario, (b) SSP2-4.5 climate scenario, (c) SSP3-7.0 climate scenario, (d) SSP5-8.5 climate scenario. This base map was directly obtained from the calculation of MaxEnt with the bioclimatic data from WorldClim. Global bioclimate data were acquired from the WorldClim open database ([https://worldclim.org](https://worldclim.org/)). The locations of species occurrence were collected from open databases: GBIF ([http://www.gbif.org](http://www.gbif.org/)), the Chinese Virtual Herbarium (CVH, <https://www.cvh.ac.cn/>), the National Specimen Information Infrastructure (NSII, http://nsii.org.cn/2017/AboutUs.php), and Chinese Field Herbarium (CFH, <https://cfh.ac.cn/>). The species distribution model was conducted with MaxEnt (Version 3.4.4, <http://biodiversityinformatics.amnh.org/open_source/maxent/>), and results were modified with ArcGIS (version 10.7, <https://www.arcgis.com/>).
